# Cell-of-origin–specific proteomics of extracellular vesicles

**DOI:** 10.1093/pnasnexus/pgad107

**Published:** 2023-04-03

**Authors:** Sebastian Kehrloesser, Oliver Cast, Thomas S Elliott, Russell J Ernst, Anne C Machel, Jia-Xuan Chen, Jason W Chin, Martin L Miller

**Affiliations:** Cancer Research UK Cambridge Institute, University of Cambridge, Li Ka Shing Centre, Robinson Way, Cambridge CB2 0RE, UK; Cancer Research UK Cambridge Institute, University of Cambridge, Li Ka Shing Centre, Robinson Way, Cambridge CB2 0RE, UK; Medical Research Council Laboratory of Molecular Biology, Francis Crick Ave, Cambridge CB2 0QH, UK; Medical Research Council Laboratory of Molecular Biology, Francis Crick Ave, Cambridge CB2 0QH, UK; Cancer Research UK Cambridge Institute, University of Cambridge, Li Ka Shing Centre, Robinson Way, Cambridge CB2 0RE, UK; Cancer Research UK Cambridge Institute, University of Cambridge, Li Ka Shing Centre, Robinson Way, Cambridge CB2 0RE, UK; Institute of Molecular Biology, Ackermannweg 4, 55128 Mainz, Germany; Medical Research Council Laboratory of Molecular Biology, Francis Crick Ave, Cambridge CB2 0QH, UK; Cancer Research UK Cambridge Institute, University of Cambridge, Li Ka Shing Centre, Robinson Way, Cambridge CB2 0RE, UK; Oncology Data Science, Oncology R&D, AstraZeneca, 1 Francis Crick Ave, Cambridge CB2 0AA, UK

**Keywords:** extracellular vesicles, proteomics, cell-specific labeling, intercellular signaling

## Abstract

The ability to assign cellular origin to low-abundance secreted factors in extracellular vesicles (EVs) would greatly facilitate the analysis of paracrine-mediated signaling. Here, we report a method, named selective isolation of extracellular vesicles (SIEVE), which uses cell type-specific proteome labeling via stochastic orthogonal recoding of translation (SORT) to install bioorthogonal reactive groups into the proteins derived from the cells targeted for labeling. We establish the native purification of intact EVs from a target cell, via a bioorthogonal tetrazine ligation, leading to copurification of the largely unlabeled EV proteome from the same cell. SIEVE enables capture of EV proteins at levels comparable with those obtained by antibody-based methods, which capture all EVs regardless of cellular origin, and at levels 20× higher than direct capture of SORT-labeled proteins. Using proteomic analysis, we analyze nonlabeled cargo proteins of EVs and show that the enhanced sensitivity of SIEVE allows for unbiased and comprehensive analysis of EV proteins from subpopulations of cells as well as for cell-specific EV proteomics in complex coculture systems. SIEVE can be applied with high efficiency in a diverse range of existing model systems for cell–cell communication and has direct applications for cell-of-origin EV analysis and for protein biomarker discovery.

**Significance Statement**

We have developed a new method for cell-of-origin–resolved extracellular vesicle (EV) proteomics with original contributions to the field including (i) the design and synthesis of novel tetrazine compounds for bioorthogonal labeling under nondenaturing conditions, (ii) the development of new protocols of enrichment of intact EVs via bioorthogonal tetrazine ligation, and (iii) the implementation of EV proteomics and computational approaches to evaluate and benchmark cell type-specific proteomics of complex cocultures. This method has direct applications for studying paracrine-mediated signaling by EVs and for protein biomarker discovery.

## Introduction

Tissues consist of a range of different cell types, and their heterotypic interactions determine tissue organization and homeostasis. Secreted factors, such as proteins and proteins associated with extracellular vesicles (EVs), play a major role in cell–cell communication and dysfunctional paracrine-mediated signaling is a hallmark of disease (1). Understanding which cell types secrete which proteins or EVs is therefore of paramount importance in both basic biology and in clinical settings (2, 3). However, the difficulty of assigning the cellular origin of secreted proteins or EVs limits the use of mass spectrometry (MS)-based proteomics to effectively identify secreted factors from cell populations of interest (4).

Strategies for cell type-specific proteomics have been developed that combine cell-selective protein labeling and MS; in all cases, the selectivity of labeling is defined by the selective expression of a transgene in the target cells, which enables the introduction of a label into its proteins (4). Proteins within cells and compartments may be labeled post-translationally by, e.g. ascorbate peroxidase for biotinylation of proximal proteins (APEX) (5) and proximity-dependent biotin identification (BioID) (6, 7). However, these methods do not define the biosynthetic origin of proteins and therefore do not provide a direct connection between the labeled proteins and the cell's genome. Moreover, these methods have not been used to define the proteomes of cell-of-origin specific secretomes or cell-of-origin specific EVs. While a recent publication developed a new approach for cell type-specific EV proteomics through the expression of APEX2 fused to the canonical EV marker protein CD63 (8), this promising methodology is yet to be demonstrated for cell selectivity in complex settings such as coculture systems or in tissues. Several labeling methods—based on cell-specific, cotranslational labeling of proteins—can define the biosynthetic origin of proteins. These methods include Cell Type-specific labeling using Amino acid Precursors (CTAP) (9), cell type-specific variants of BioOrthogonal Non-Canonical Amino acid Tagging (BONCAT) (10), and stochastic orthogonal recoding of translation (SORT) (11).

In CTAP, stable isotope-labeled precursors of canonical amino acids are selectively converted to the corresponding amino acids upon the expression of amino acid biosynthesis enzymes in the cells of interest. The resulting isotopic amino acids are cotranslationally incorporated into the cell's proteome, which enables near-full differential proteome labeling of two cell populations in coculture labeled by heavy and light isotopes using two distinct amino acid precursors and two distinct biosynthesis enzymes. However, CTAP is limited to analyzing cell-selective proteomics and secretomics in cocultures with two different cell populations of about equal proportions.

In cell type-specific BONCAT, the cell of interest expresses a mutant version of an endogenous aminoacyl-tRNA synthetase that accommodates a noncanonical amino acid (ncAA) to enable cell type-specific proteome labeling with the ncAA. In SORT, orthogonal pyrrolysyl-tRNA synthetase/tRNA pairs, directed to sense codons, are used to label the proteome with ncAAs. For both SORT and BONCAT, the proteome is labeled substoichiometrically (∼1–2% of target codons) with ncAAs bearing bioorthogonal groups. The low level of labeling minimizes the effects of ncAAs on protein function (11, 12), and the bioorthogonal groups enable the covalent enrichment of low-abundance labeled proteins from cells of interest in complex samples. SORT and versions of BONCAT have been applied to interrogate distinct subpopulations of cells in complex environments, including specific cell types in *C. elegans*, *D. melanogaster*, and the brains of live mice (11, 13–16).

While cell-selective labeling approaches, or variants thereof, have been widely applied to interrogate the dynamics of the proteome in complex cocultures or in situ in tissues (11, 14–19), few reports have focused on using these methods for analyzing secreted proteins (9, 20, 21) and to our knowledge no method has been developed for cell-of-origin specific labeling and analysis of the EV proteome. Here, we demonstrate that using SORT to label the proteome in a desired cell type leads to EVs containing proteins biosynthesized in that cell type. Through covalent capture of SORT-labeled proteins on the surface of EVs, we selectively enrich EVs enabling proteomic analysis of the (largely) unlabeled contents of the EVs originating from the desired cell type. We demonstrate the utility of this approach for detecting and analyzing the EV proteome from subpopulations of cells in complex coculture systems.

## Results

### SORT labeling enables capture of intact EVs

**Fig. 1.**
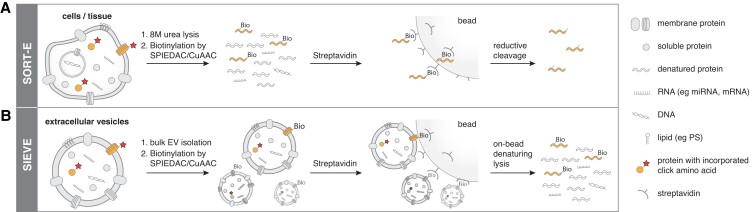
Bioorthogonal protein labeling for cell-of-origin selective enrichment of EVs with SIEVE. A) In SORT-E, cells are engineered to express orthogonal tRNA synthetase/tRNA pairs that introduce ncAAs at a low incorporation frequency in the proteome. The use of ncAAs, such as alkyne or strained alkene amino acids (click amino acid), allows for subsequent bioorthogonal labeling for imaging or enrichment of ncAA-containing proteins by copper-catalyzed azide–alkyne cycloaddition (CuAAC) or strain-promoted inverse electron-demand Diels–Alder cycloaddition (SPIEDAC). B) SIEVE applies SORT-E in nondenaturing conditions to capture intact EVs on beads by labeling of exposed proteins containing at least one ncAA and enables copurification of nonlabeled EV cargo including proteins, lipids, and nucleic acids.

To develop selective isolation of extracellular vesicles (SIEVE) for cell-of-origin selective EV proteomics, we applied SORT to stochastically label the proteome of target cells including EV surface proteins for subsequent covalent capture and interrogation of EV cargo (Fig. 1). Mouse fibroblast L cells secrete large amounts of EVs (22), so we targeted these cells for our studies. To enable SORT labeling of the proteome, we created piggyBac constructs (23), expressing the pyrrolysyl tRNA synthetase (*Pyl*RS) and tRNAs targeting serine sense codons (*Pyl*T_GCU_), and integrated these into the genome of L cells. Upon the addition of ncAAs bearing bioorthogonal groups such as cyclopropene-l-lysine ([((methylcycloprop-2-en-1-yl)methoxy)carbonyl]-L-lysine) (CypK) and alkyne-l-lysine (Nε-(propargyloxycarbonyl)-l-lysine) (AlkK) that are substrates for PylRS, we observed proteome labeling in L cells. We also integrated a human version of the exosome marker CD81 (hCD81) *C*-terminally fused to nanoluc luciferase to enable sensitive quantification of hCD81-positive EVs using a luciferase assay. The resulting cell line, L cell_SORT_GCU_-hCD81_Nluc_, was used for EV purification.

**Fig. 2.**
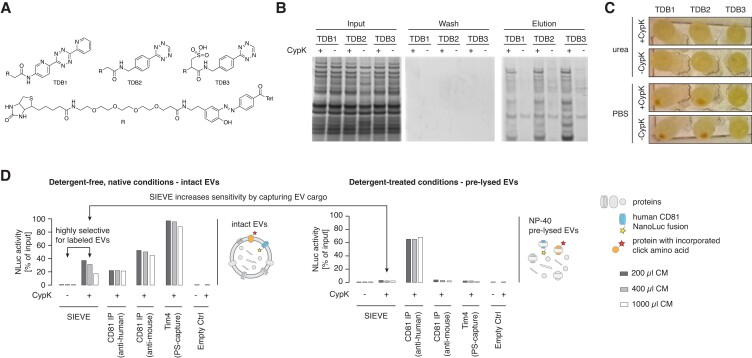
Optimization of tetrazine ligation under nondenaturing conditions enables SIEVE-based enrichment of intact EVs with high recovery rates. A) Chemical structures of tetrazine-diazobenzene-biotin (TDB) based on TDB1 (12) and derivatives with decreased hydrophobicity for increased water solubility. B) Expression and purification of soluble TDB1-3 in *E. coli*—lysate labeling and enrichment are shown under native conditions (PBS) and detected with Sypro ruby. C) Photograph of precipitates after the labeling reaction. D) EVs were preenriched using ultrafiltration of cell culture supernatant from L cell_SORT_GCU_-hCD81_NLuc_ grown in the presence or absence of 0.25 mm CypK. Enriched EVs were then further purified in a plate-based assay using the method indicated under detergent-free, native conditions (left panel) or after pretreatment with NP-40 to lyse EVs but keep protein structures and functions intact (right panel). Recovery efficiency is shown as retained luminescence intensity as a percentage of input luminescence (NLuc activity). Note that the observed decrease in capture efficiency with increasing culture medium (CM) volume is mainly due to competition with increasing concentrations of free TDB compound during the binding step that could not be removed in this experimental setup. Binding capacity of the immobilized streptavidin also suppressed recovery rates at higher EV concentrations, as demonstrated by decreasing recovery rates with increasing CM volumes using an antibody targeting endogenous murine CD81 as well as PS-capture. Bar colors indicate the corresponding original cell culture medium (CM) volume before preenrichment (*n* = 1 sample from three different CM volumes).

Next, we aimed to capture EVs from L cell_SORT_GCU_-hCD81_Nluc_ cells in which the proteome had been SORT labeled upon the addition of CypK. Under the native conditions required to purify intact EVs, the tetrazine-diazobenzene-biotin (TDB) compounds that we previously used to capture CypK-labeled proteins [via a strain-promoted inverse electron demand Diels-Alder cycloaddition (SPIEDAC)] in SORT-E were insoluble and precipitated (Fig. 2A–C). We therefore designed and synthesized a soluble TDB compound, TDB3 (Fig. S1, Supplementary Material); this compound enabled the efficient capture of EVs from L cell_SORT_GCU_-hCD81_NLuc_ grown in the presence of CypK under native conditions (Fig. 2D, left panel).

We assessed the EV recovery efficiency using the luciferase assay, which is based on the presence of hCD81 *C*-terminally fused to nanoluc in EVs. This assay demonstrated that SIEVE captures up to 40% of the nanoluc positive EVs using TDB3-mediated SIEVE, and control experiments confirmed that EV capture is CypK dependent (Fig. 2D). We used the same luciferase assay to compare SIEVE with commonly used affinity isolation methods that do not distinguish the EV's cell of origin, i.e. immunoprecipitation with biotinylated antibodies targeting human CD81 (CD81 IP), endogenous mouse CD81, or lipid affinity purification with a biotinylated version of the phosphatidylserine (PS) binding protein Tim4 (24). The efficiency of capture was similar to antibody-based capture and 2–2.5 times less than PS-capture (Fig. 2D).

To demonstrate the value of capturing intact EVs, over direct capture of labeled proteins from a defined cell-of-origin (20, 21), we lysed the EVs from L cell_SORT_GCU_-hCD81_NLuc_ grown in the presence of CypK with a mild nondenaturing detergent (0.5% NP-40). Purification with an anti-mouse CD81 antibody or Tim4 (PS-capture) did not copurify luciferase activity, whereas purification with an anti-human CD81 antibody captured robust luciferase activity; these experiments demonstrate that the EVs are lysed. Capture of proteins from EV lysates with TBD3 led to a luciferase signal that barely exceeded that of the negative control from L cell_SORT_GCU_-hCD81_NLuc_ cells that were not provided with CypK. The luciferase activity isolated from intact EVs, via SIEVE, is 20 times higher than the activity captured via direct TBD3-mediated purification of labeled proteins from the lysed EVs. Our data demonstrate that SIEVE enables superior recovery of EV proteins compared with methods that rely on directly capturing of labeled proteins from a specific cell type.

### SIEVE preferentially enriches for EV proteins

**Fig. 3.**
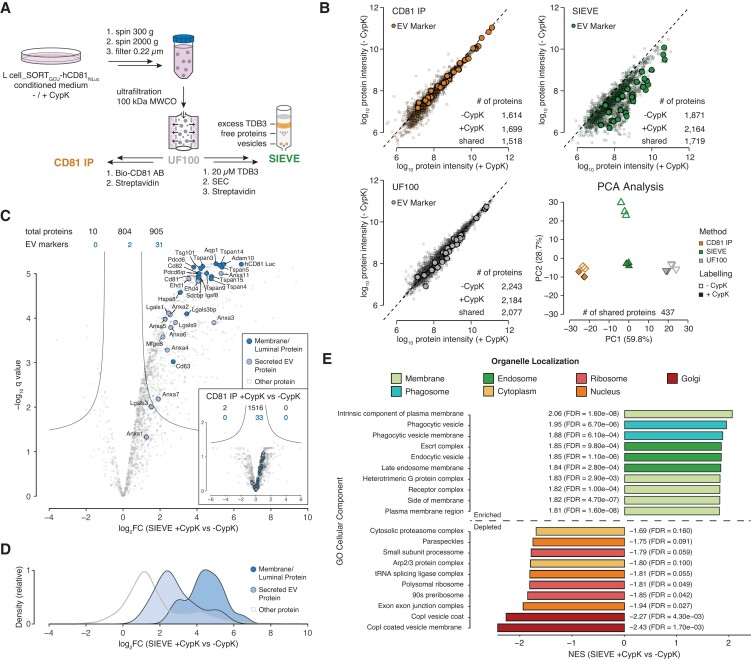
SIEVE preferentially enriches for EV-associated membrane and EV cargo proteins. A) Schematics of the experimental setup indicating at which step of the purification process samples were subjected to MS analysis as indicated by the sample names CD81 IP, UF100 (ultrafiltration with 100-kDa filter), or SIEVE. B) Scatter plots of mean protein abundances across replicates comparing isolation of CypK^−^ and CypK^+^ EVs for each method and PCA analysis comparing the intersection of proteins identified across all conditions. EV marker proteins are highlighted (*n* = 3 for all samples except *n* = 2 for CD81 IP_CypK^−^, number of proteins identified in each condition indicated). C) Differential abundance analysis of SIEVE enrichment of CypK^+^ EV proteins over CypK^−^ control expressed as log_2_ fold change (log_2_FC) and significance of change using multiple hypothesis corrected *P* value from two-sided *t*-tests (*q* value). Inset represents the corresponding analysis for enrichment with CD81 IP. Lines represent the cut-off curve for significance (S0 = 1, *q* < 0.05). EV marker proteins are highlighted as blue circles with luminal/membrane proteins in dark blue and EV-associated, secreted proteins in light blue (*n* = 3 for both conditions, number of proteins identified is indicated). D) Marginal density plot based on log_2_FC distribution of the data points in the three categories presented in panel C. E) Normalized enrichment scores (NES) of preranked GSEA of differentially quantified proteins in SIEVE CypK^+^ vs CypK^−^ control (log_2_FC) using cellular localization gene sets. The top 10 enriched and depleted gene sets are shown and categorized by manual annotation of organelles.

Next, we investigated whether SIEVE allows for selective enrichment of EV-associated membrane and cargo proteins using unbiased MS analysis. To test this, we cultured L cell_SORT_GCU_-hCD81_NLuc_ cells to 80–90% confluency and for 48 h in serum-free medium prior to harvesting EVs. We then performed proteomic analysis of SIEVE-purified EVs compared with generic EV enrichment by ultrafiltration alone or followed by additional isolation by immuno-precipitation targeting the EV marker CD81 using an anti-mouse CD81 antibody (Fig. 3A). Enrichment of CypK^−^ and CypK^+^ EVs by either ultrafiltration or antibody isolation showed that the measured EV proteomes remain unchanged by the presence of 0.25 mm CypK as indicated by similar protein abundances between the two conditions with neither method being able to distinguish CypK labeled from nonlabeled EVs (Fig. 3B, lower left and upper left scatter plots). SIEVE, however, resulted in higher mean intensities and protein identification rates for the labeled samples (Fig. 3B, green scatter plot) and was the only method showing a clear separation of labeled and unlabeled samples in a principal component analysis (PCA) using the intersection of identified proteins across all samples (Fig. 3B, lower right quadrant).

To further interrogate specific EV protein components, we analyzed a set of proteins known to be associated with either the membrane or lumen of EVs or secreted EV-associated proteins (Table S1, Supplementary Material) (1, 3, 25). The majority of these high confidence EV marker proteins (31 of 33) was significantly enriched in CypK-labeled samples purified by SIEVE compared with the nonlabeled control, with membrane and luminal proteins showing stronger enrichment than secreted EV-associated proteins (Fig. 3C and D). In contrast, no EV marker protein was significantly enriched comparing CD81-enriched CypK^+^ and CypK^−^ EV proteins (Fig. 3C inset, Table S2, Supplementary Material). Furthermore, SIEVE can effectively be used to distinguish EV-specific proteins from unspecific background proteins as more than half (905 of 1,719) of the total number of proteins identified were significantly enriched in the CypK^+^ condition with many of these proteins potentially representing novel EV-associated proteins. In particular, we found that SIEVE clearly distinguished background binding proteins sticking to column material (sometimes referred to as “contaminants,” Table S1, Supplementary Material) from EV markers comparing CypK^+^ vs CypK^−^ labeled conditions compared with the other two EV approaches tested (Fig. S2, Supplementary Material). The observation that SIEVE enriches for EV proteins further was supported by performing an unbiased preranked gene set enrichment analysis (GSEA) interrogating cellular component gene sets (26). This analysis showed that the SIEVE-isolated proteome has a strong enrichment of integral and membrane-associated proteins as well as endosomal and more specifically Escort complex proteins, which are involved in the exosome biogenesis process (Fig. 3E) (1). In contrast, abundant cytoplasmic, nuclear, or ribosomal proteins were strongly depleted (Fig. 3E), further demonstrating preferential isolation of intact EVs and coisolation of nonlabeled cargo proteins. Moreover, we observed no significant abundance changes of EV marker proteins between CypK^+^ and CypK^−^ conditions of L cell_SORT_GCU_-hCD81_Nluc_ cells when EVs were enriched with CD81 (Fig. 3C inset) or UF100 (Fig. S2B, Supplementary Material) indicating that incorporation of ncAAs in itself has limited perturbing effects on the EV proteome. A list of proteins identified with the three different EV enrichment methods can be found in Table S2, Supplementary Material.

### Proteomic interrogation of low abundant EV subpopulations enabled by SIEVE

**Fig. 4.**
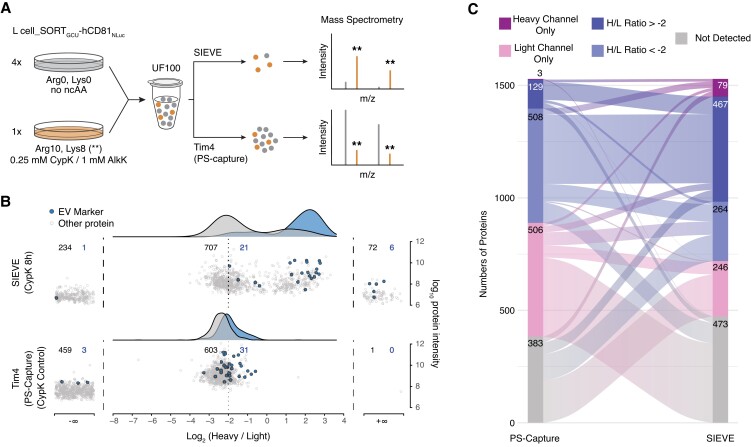
Low-abundant EV subpopulations are selectively enriched by SIEVE and analyzed by MS. A) Schematics of experimental setup mixing L cell_SORT_GCU_-hCD81_NLuc_ EVs isolated by ultrafiltration (UF100) from light or heavy SILAC labeled L cells and the expected effect on the heavy to light (H/L) ratio as illustrated for two peptides analyzed by MS after PS-capture and SIEVE, respectively. Mass to charge (*m*/*z*) peaks for light peptide species are shown in grey, and peaks for Arg10/Lys8 heavy-labeled peptides are shown in orange and highlighted by asterisks. B) Distribution of the H/L ratio for all quantified proteins comparing isolation by SIEVE (top panel) and generic isolation by Tim4 (PS-capture, bottom panel). EV marker proteins are highlighted in blue, and density plots of H/L ratio distributions of marker and nonmarker proteins are shown on top using the same color scheme. The number of quantified proteins are given for shared, light only, and heavy only populations (*n* = 1,041 and 1,097 total quantified proteins for one representative SIEVE and PS-capture, respectively). C) Sankey diagram of data from B, showing the relationship between proteins identified by the two methods categorized into five discrete bins as indicated.

For SIEVE to be useful for studying EV mediated cell–cell communication, it is required that SIEVE can specifically isolate labeled target EVs from complex mixtures. In order to assess selective enrichment of low abundant labeled EVs in a background of unlabeled EVs, we performed SIEVE on a 1:4 mixture of EVs isolated from SILAC heavy-labeled (H) and CypK^+^ labeled L cells and SILAC light (L) and nonlabeled (CypK^−^) L cells and compared this with generic isolation using PS-capture with Tim4 (Fig. 4A). Purification based on PS-capture resulted in the majority of proteins showing a log_2_ heavy to light (H/L) ratio of −2, corresponding to the initial mixing ratio, irrespective of whether or not a given protein was classified as an EV marker (Fig. 4B, lower plot). In addition, the generic PS-capture produced a fraction of proteins detected only in the light channel—consistent with that the majority of EVs in the mixture originating from the SILAC light-labeled L cell population. In contrast, SIEVE resulted in a clear and significant shift of EV marker proteins to higher H/L ratios and a bimodal distribution of non-EV marker proteins; this demonstrates selective retention of labeled EVs, with detection of preferentially heavy-labeled and heavy channel only proteins from a subpopulation of EVs (Fig. 4B, upper plot). Comparing the two methods by discretizing proteins by their light and heavy status further supported SIEVE-based enrichment as nearly half of the proteins not detected by PS-capture (143 of 383) could be detected as a heavy-labeled protein by having a H/L ratio or being detected in the heavy channel only (Fig. 4C). In addition, depletion of nonlabeled background EVs with SIEVE was further supported by the observation that the number of proteins only detected in the light channel was reduced by >50% from 506 with PS-capture to 246 with SIEVE (Fig. 4C). Testing SIEVE using azide alkyne chemistry for EV enrichment based on AlkK and copper-dependent azide alkyne cycloaddition (Fig. S3, Supplementary Material) and repeating the SILAC experiment resulted in nearly identical enrichment patterns showing the robustness of our approach (Fig. S4, Supplementary Material).

### SIEVE enables cell-of-origin EV proteome analysis from complex coculture systems

**Fig. 5.**
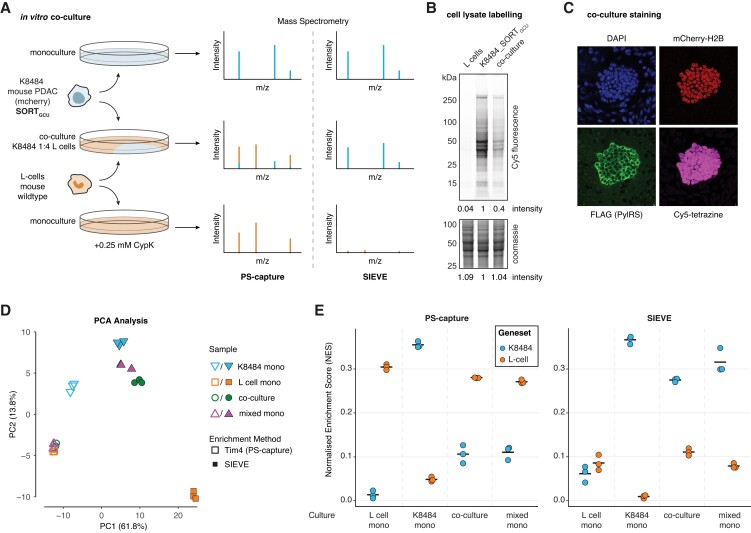
Cell type-specific EVs are selectively isolated from cocultures allowing for cell-of-origin resolved EV proteomics. A) Schematics of experimental setup comparing proteomic analysis of individual monocultures as well as a 1:4 coculture of the SORT-engineered KPC PDAC cell line K8484_SORT_GCU_ and L cell fibroblasts after purification by either PS-capture or SIEVE. A direct mixture of monoculture supernatants (mixed mono) was used as an additional control for the coculture conditions (not shown). B) Cell lysate labeling after 4 days of (co)culture. Lysates were labeled with Cy5-tetrazine for in-gel fluorescence before counterstaining with Coomassie for total protein load. Lane intensities have been determined by densitometry and normalized to the K8484_SORT_GCU_ measures. C) Confocal microscopy images of cocultures showing a nuclear stain with DAPI staining both cell lines and highlighting the K8484_SORT_GCU_ cells by detecting mCherry and staining for FLAG tag (*Pyl*RS). Slides were further labeled with Cy5-tetrazine for incorporation of CypK. D) PCA of EV proteomes based on PS- and SIEVE-captured EVs from mono- and coconditions using proteins with a SILAC heavy/light ratio > 0 using data from Fig. 4B (*n* = 265 proteins, *n* = 24 conditions). E) Normalized enrichment score (NES) of ssGSEA analysis of EV proteome data from D using two gene sets specific to either K8484 or L cells (Fig. S5B, Supplementary Material). Mean NES for each condition is indicated by a bar.

While the SILAC experiment clearly demonstrated that SIEVE is able to efficiently recover a low abundant EV subpopulation and thus increase sensitivity for MS analysis of specific EV target populations, mixing of isotopically labeled EVs does not reflect the sample conditions most likely obtained from biologically relevant coculture or in vivo models. In order to assess the capability of SIEVE for the analysis of these more complex systems, we engineered the murine pancreatic cancer cell line K8484 (27) derived from the KPC (Trp53^R172H^, Kras^G12D^, Pdx1-Cre) mouse model (28) to express the SORT machinery and mCherry-H2B (K8484_SORT_GCU_). K8484_SORT_GCU_ cells were seeded in a 1:4 ratio with wild-type murine fibroblasts (L cells) and grown as a 2D coculture for 72 h and an additional 24 h after exchanging media to serum-free conditions before harvesting lysate and EV samples from cocultures and separately from both monocultures (Fig. 5A). Efficient incorporation of CypK was demonstrated by lysate labeling with a Cy5-tetrazine compound followed by in-gel fluorescence with the K8484_SORT_GCU_ monoculture and the coculture being positive and L cells being negative as expected (Fig. 5B). Furthermore, fluorescence microscopy of fixed cocultures seeded at the same time showed that Cy5-tetrazine staining colocalized with K8484_SORT_GCU_ specific markers *Pyl*RS and mCherry-H2B (Fig. 5C). Both methods showed high selectivity of incorporation, and quantification of in-gel fluorescence indicated that final cell ratios after 4 days of coculture reached 1:2.5 (K8484_SORT_GCU_:L cells).

To assess specificity of SIEVE compared with generic EV isolation with PS-capture in this fibroblast and cancer cell coculture, we compared triplicates of PS (Tim4)- and SIEVE (SPIEDAC)-purified EV samples of four conditions consisting of both monocultures, the K8484_SORT_GCU_ and L cells coculture, as well as mixed monoculture samples admixing EVs from each cell lines in a 1:4 ratio to match the seeding ratio of the coculture. We first explored overall differences in protein abundances between the EV proteomes of the mono- and cocultures of the two mouse cell lines. To focus on EV-associated proteins and effectively remove background binders irrespective of the cell line analyzed, we restricted the analysis to putative EV proteins as defined by having a H/L log ratio > 0 in the previous SILAC experiment (Fig. 4B). PCA of this EV-associated proteome (*n* = 265) across the different conditions showed that the majority of the variation between samples (61.8% in PC1) could be attributed to the different enrichment methods (Fig. 5D). For the PS samples, K8484_SORT_GCU_ monocultures clustered separately, while L cell monocultures clustered together with the mixed monoculture and coculture conditions, likely reflecting the original mixing ratios and that L cells secrete relatively large amounts of EVs. For the SIEVE samples, however, L cell monocultures appeared as outliers as these samples effectively represent background binders (Fig. S5A, Table S3, Supplementary Material). As expected, K8484_SORT_GCU_ monocultures clustered closer to mixed monoculture and coculture conditions, underlining the ability of SIEVE to selectively isolate K8484_SORT_GCU_-derived EVs from the mixed conditions.

Using GSEA, we next sought to effectively benchmark cell selectivity of SIEVE given the complexity that EV proteins, like other proteins, are not necessarily unique to a cell type. Rather, it is the relative abundance between proteins of a given cell type that can be used as a signature of cell-of-origin. To generate two lists of EV proteins that could be used as a signature for each cell line, we first performed differential expression analysis of the EV proteomes of PS-isolated EV samples of K8484_SORT_GCU_ cells against L cells identifying EV proteins that were significantly different between the two cell lines (Fig. S5B, Table S4, Supplementary Material). We then applied single sample GSEA (ssGSEA) to determine the enrichment of each of these lists of proteins in each condition using the EV-associated proteome as defined before. All monoculture conditions showed the expected enrichment pattern as the normalized enrichment score (NES) of each cell type-associated gene set was high for the matched cell line and low for the other cell line, respectively (Fig. 5E). As expected, the only exception from this pattern was the low L cell NES for the SIEVE-enriched L cell monocultures as these samples consist mainly of background binders as previously noted. Similarly, the mixed conditions also showed the expected enrichment pattern: SIEVE-isolated mixed conditions showed strong enrichment of the K8484 over L cell proteins, while PS-isolated mixed conditions showed the opposite pattern reflecting the original 1:4 mixing ratio (Fig. 5E). These results demonstrate that SIEVE enables cell-of-origin resolved EV proteomics from a subpopulation of cells in coculture with another cell type.

## Discussion

The importance of EVs in cell–cell communication and the presence of EVs in peripheral fluids as a valuable source of disease biomarkers have fueled the recent interests in both basic and translational aspects of EV biology (3, 29). We have developed a method that combines SORT with enrichment of intact EVs for unbiased, systematic analysis of EV content from a cell population of interest. The key features of SIEVE are that (i) the polyvalent display of ncAAs on the EV surface makes capture efficient and (ii) because capture is based on proteome labeling rather than specific markers, our approach is agnostic with respect to EV subtype and content—offering better opportunity for unbiased discovery than with methods that capture EVs based on specific proteins.

The current SORT approach with enrichment (SORT-E) specifically isolates SORT-labeled proteins under harsh denaturing conditions followed by selective release through reductive cleavage of the biotinylation reagent (12). While SORT-E efficiently suppresses nonspecific background binding of nonlabeled species enabling highly selective isolation, the low labeling frequency required for a perturbation-free labeling also means that at most ∼1% of the target proteome can be recovered (11, 12). Therefore, the amount of starting material required can be a limiting factor for the proteomic analysis of low abundant protein species, such as secreted factors. However, since incorporation probability is proportional to the number of SORT targeted codons and larger entities have a higher chance of incorporating at least one ncAA, we reasoned that capturing intact vesicles with at least one surface exposed ncAA would allow copurification of nonlabeled cargo proteins and even nonprotein components such as lipids and nucleic acids.

We show how the principle of capturing large entities such as EVs using bioorthogonal labeling with SIEVE has several advantages: (i) by effectively expanding the total number of SORT targeted codons to include all surface exposed EV proteins, SIEVE overcomes sensitivity issues and enhances recovery efficiency 20× compared with standard bioorthogonal labeling of single molecules; (ii) expressing the SORT machinery in target cells enables cell-selective EV enrichment from subpopulations of cells in benchmark mixture conditions as well as in complex coculture systems; (iii) comparing SORT-expressing cells with and without supplement of ncAAs using the SIEVE isolation protocols allows to effectively distinguish true EV proteins from background binders, which is challenging with standard EV isolation approaches such as ultrafiltration, centrifugation, and affinity purification that do not have similar controls; and (iv) the stochastic nature of SORT labeling enables random labeling of EVs irrespective of their intracellular origin (endosomal, plasma membrane, or other origin) without the need of tagging specific EV components, which could affect biogenesis or bias toward specific EV subsets (exosomes, microvesicles, or others). Together, these advantages make SIEVE a powerful approach for unbiased, cell-selective analysis of a variety of vesicle types and their cargo. The main limitations of SIEVE, however, are the requirements of expressing the SORT machinery and the use of ncAAs, which may limit its use in certain model systems and makes it incompatible with analysis of primary human tissues and fluids.

In this work, we used a mouse fibroblast and PDAC coculture experiment to evaluate the cell type-specificity of SIEVE. We applied ssGSEA to evaluate the ability of SIEVE to specifically interrogate the EV proteome from PDAC cells without the use of common benchmarking approaches such SILAC, CTAP, or by peptide sequence differences between human and mouse (9). With this mouse coculture setup, we demonstrated selective enrichment of EV proteins from a subpopulation of cells using SIEVE (PDAC cells), while proteome analysis of generic EV isolation using PS-capture reflected the major cell population (L cells). This shows the relative abundance of secreted EV proteins from a given cell type that can be used as a signature of cell-of-origin. We anticipate that a similar experimental–computational approach can be applied in other complex model systems, such as in mice, to evaluate the ability of SIEVE or other methods to analyze EVs and their content from a specific cell population of interest.

The principle of applying bioorthogonal protein labeling to perform proteomics of cell-of-interest in mouse tissues has been demonstrated in multiple settings (11, 13–16). Future work will develop SIEVE for in vivo analysis by developing protocols to capture EVs based on labeling with AlkK, which can be synthesized inexpensively and fed to animals in the drinking water for in vivo protein labeling in tissues as we have previously demonstrated (12, 15). MS-based proteomics of complex fluids is hampered by the large dynamic range of proteins in cultured growth medium or blood and the presence of abundant background proteins like albumin (2, 30). This makes comprehensive and unbiased analysis of the secretome of specific cell types in animal models or coculture systems challenging. In addition, the difficulty of assigning the cellular origin of secreted proteins or EVs limits the use of proteomics to effectively identify secreted factors from cell populations of interest. Applying SIEVE for in vivo models has the potential to overcome both issues of sample complexity and assigning EVs to their cell-of-origin. In addition, future work will apply SIEVE and expand the analysis of other components of EVs, such as lipids and nucleic acids.

We believe that the possibility of applying SIEVE to the wealth of existing coculture systems and preclinical mouse models will facilitate the investigation of paracrine-mediated signaling and discovery of disease-related biomarkers. Given the major importance of protein-based biomarkers for monitoring human health and their potential use for early disease detection (2, 31, 32), new experimental approaches, such as SIEVE, that enables cell-of-origin resolved secretomics are needed to facilitate the discovery of candidate biomarkers and the interrogation of cell–cell communication via secreted factors.

## Materials and methods

### Chemical syntheses: general methods

Synthesis of diazobenzene carboxylic acid **1** (Scheme S1, Fig. S1, Supplementary Material) was previously reported (12). All chemicals and solvents were purchased from Sigma-Aldrich, Alfa Aesar, or Fisher Scientific and used without further purification unless otherwise stated. Qualitative analysis by thin layer chromatography (TLC) was performed on aluminum sheets coated with silica (Merck TLC 60F-254). The spots were visualized under short wavelength ultraviolet lamp (254 nm) or stained with basic, aqueous potassium permanganate, ethanolic ninhydrin, or vanillin. Flash column chromatography was performed with specified solvent systems on silica gel 60 (mesh 230–400). Liquid chromatography tandem mass spectrometry (LC–MS) analysis was performed on Agilent 1200 machine. The solvents used consisted of 0.2% formic acid in water (buffer A) and 0.2% formic acid in acetonitrile (buffer B). LC was performed using Phenomenex Jupiter C18 column (150 × 2 mm, 5 µm) and monitored using variable wavelengths. Retention times (Rt) are recorded to a nearest 0.1 min and *m*/*z* ratio to nearest 0.01 mass units. The following program was used for small molecule LC gradient: 0–1 min (A:B 10:90–10:90, 0.3 mL/min), 1–8 min (A:B 10:90–90:10, 0.3 mL/min), 8–10 min (A:B 90:10–90:10, 0.3 mL/min), and 10–12 min (A:B 90:10–10:90, 0.3 mL/min). MS analysis following LC was carried out in ESI mode on an Agilent 6130 single quadrupole spectrometer and recorded in both positive and negative ion modes.

#### Methyl (*E*)-(4-((5-(2-((*tert*-butoxycarbonyl)amino)ethyl)-2-hydroxyphenyl)diazenyl)benzoyl)glycinate

Diazobenzene carboxylic acid 1 ((12), 100 mg, 0.25 mmol, 1 eq) was dissolved in CH_2_Cl_2_ (1 mL) and DMF (0.5 mL), to which was added H-Gly-OMe·HCl (35 mg, 0.29 mmol, 1.1 eq), followed by EDCI (54 mg, 0.29 mmol, 1.1 eq), HOBT (38 mg, 0.29 mmol, 1.1 eq), and DMAP (cat.). The reaction mixture was stirred at room temperature (RT) for 18 h and adjudged complete at this time by LC–MS analysis. The reaction mixture was diluted with EtOAc (10 mL) and washed with 1 m HCl (2 × 10 mL) and then brine (10 mL). The organics were dried (Na_2_SO_4_), filtered, and concentrated under vacuum to give a crude gum. The crude material was purified by silica gel column chromatography eluting EtOAc/hexane gradient (20/80 then 40/60 then 80/20). The product fractions were combined and concentrated under vacuum to give methyl (*E*)-(4-((5-(2-((*tert*-butoxycarbonyl)amino)ethyl)-2-hydroxyphenyl)diazenyl)benzoyl)glycinate as a dark orange gum (22 mg, 20% yield). The corresponding m/z as detected by low-resolution mass spectrometry (LRMS) via electrospray ionization is the following: *m*/*z* (ES+) 457 [M + H]^+^, *m*/*z* (ES−) 455 [M − H]^−^.

#### (*E*)-(4-((5-(2-((*tert*-Butoxycarbonyl)amino)ethyl)-2-hydroxyphenyl)diazenyl)benzoyl)glycine **2a**

Methyl (*E*)-(4-((5-(2-((*tert*-butoxycarbonyl)amino)ethyl)-2-hydroxyphenyl)diazenyl)benzoyl)glycinate (22 mg, 48 µmol, 1 eq) was dissolved in THF/H_2_O (3:1, 2 mL), to which was added LiOH·H_2_O (1.3 mg, 53 µmol, 1.1 eq). The reaction mixture was stirred at RT for 1 h and adjudged complete at this time by LC–MS analysis. The reaction mixture was acidified with 1 m HCl (10 mL) and extracted with EtOAc (3 × 10 mL), and the combined organics were then washed with 1 M HCl (2 × 10 mL) and then brine (10 mL). The organics were dried (Na_2_SO_4_), filtered, and concentrated under vacuum to give crude **2a** (17 mg, quantitative yield). The material was used without further purification. The corresponding m/z as detected by LRMS via electrospray ionization is the following: *m*/*z* (ES+) 443 [M + H]^+^, *m*/*z* (ES−) 441 [M − H]^−^.

#### *tert*-Butyl (*E*)-(3-((4-((2-((4-(1,2,4,5-tetrazin-3-yl)benzyl)amino)-2-oxoethyl)carbamoyl)phenyl)diazenyl)-4-hydroxyphenethyl)carbamate **3a**

Crude **2a** (17 mg, 25 µmol, 1 eq) was dissolved in CH_2_Cl_2_ (2 mL) and DMF (0.5 mL), to which was added (4-(1,2,4,5-tetrazin-3-yl)phenyl)methanamine hydrochloride (16 mg, 72 µmol, 1.5 eq, Sigma), followed by EDCI (14 mg, 72 µmol, 1.5 eq) and DMAP (cat.). The reaction mixture was stirred at RT for 1 h and adjudged complete at this time by LC–MS analysis. The reaction mixture was diluted with EtOAc (20 mL) and washed with 1 m HCl (2 × 10 mL) and then brine (10 mL). The organics were dried (Na_2_SO_4_), filtered, and concentrated under vacuum. This gave the desired product **3a** as a red/orange solid that was used without further purification. The corresponding m/z as detected by LRMS via electrospray ionization is the following: *m*/*z* (ES+) 612 [M + H]^+^, *m*/*z* (ES−) 610 [M − H]^−^.

#### (*E*)-*N*-(2-((4-(1,2,4,5-Tetrazin-3-yl)benzyl)amino)-2-oxoethyl)-4-((5-(2-aminoethyl)-2-hydroxyphenyl)diazenyl)benzamide

Crude **3a** (∼48 µmol, 1 eq) was suspended in CH_2_Cl_2_ (2 mL), to which was added TFA (2 mL) at RT, forming a red/orange solution. LC–MS analysis after 30 min showed complete boc deprotection. The reaction was therefore concentrated to dryness by passing a stream of nitrogen over the reaction, giving the crude amine as a red/orange gum that was used directly without further purification. The corresponding m/z as detected by LRMS via electrospray ionization is the following: *m*/*z* (ES+) 512 [M + H]^+^, *m*/*z* (ES−) 510 [M − H]^−^.

#### Tetrazine-diazobenzene-biotin TDB2

Crude amine (∼48 µmol, 1 eq) was dissolved in DMF (2 mL), to which was added NHS-PEG4-Biotin (53 mg, 53 µmol, 1.1 eq, Thermo Scientific EZ-Link NHS-PEG4-Biotin—21363) and Hünig's base (18 µL, 105 µmol, 2.2 eq). The reaction was monitored by LC–MS analysis and was adjudged complete after 1 h. The reaction mixture was directly purified by semipreparative HPLC (10–90% MeCN in H_2_O over a 35-min gradient at 4 mL min^−1^ using Phenomenex Luna, 5µ, C18, 100 Å column). The product fractions were combined and freeze dried to give **TDB2** (7 mg, 15% yield over three steps) as a red/orange powder. The corresponding m/z as detected by LRMS via electrospray ionization is the following: *m*/*z* (ES+) 986 [M + H]^+^, *m*/*z* (ES−) 984 [M − H]^−^.

#### 2,5-Dioxopyrrolidin-1-yl (*E*)-4-((5-(2-((*tert*-butoxycarbonyl)amino)ethyl)-2-hydroxyphenyl)diazenyl)benzoate

Diazobenzene carboxylic acid **1** ((12), 50 mg, 0.13 mmol, 1 eq) was dissolved in DMF (1 mL), to which was added *N*-hydroxysuccinimide (18.1 mg, 0.16 mmol, 1.2 eq), followed by EDCI (30 mg, 0.16 mmol, 1.2 eq) and DMAP (8 mg, 0.07 mmol, 0.5 eq). The reaction mixture was stirred at RT for 2 h and adjudged complete at this time by LC–MS analysis. The reaction mixture was diluted with EtOAc (10 mL) and washed with 1 m HCl (2 × 10 mL) and then brine (10 mL). The organics were dried (Na_2_SO_4_), filtered, and concentrated under vacuum to give a crude gum. The crude material was purified by silica gel column chromatography eluting EtOAc/hexane (40/60). The product fractions were combined and concentrated under vacuum to give the activated ester as a dark orange gum (30 mg, 48% yield). The corresponding m/z as detected by LRMS via electrospray ionization is the following: *m*/*z* (ES+) 483 [M + H]^+^, *m*/*z* (ES−) 481 [M − H]^−^.

#### (*E*)-(4-((5-(2-((*tert*-Butoxycarbonyl)amino)ethyl)-2-hydroxyphenyl)diazenyl)benzoyl)(sulfo)alanine **2b**

Activated ester (50 mg, 104 µmol, 1 eq) was dissolved in DMF (2 mL), to which was added l-cysteic acid monohydrate (20 mg, 114 µmol, 1.1 eq) followed by Hünig's base (54 µL, 312 mmol, 3 eq). The reaction was stirred at RT for 2 h and was adjudged complete by LC–MS analysis after this time. The crude reaction mixture was acidified with 1 m HCl (10 mL) then extracted with 3:1 CHCl_3_/IPA (3 × 10 mL). The organics were dried (Na_2_SO_4_), filtered, and concentrated under vacuum to give **2b** as a crude gum that was used without further purification. The corresponding m/z as detected by LRMS via electrospray ionization is the following: *m*/*z* (ES−) 535 [M − H]^−^.

#### (*E*)-3-((4-(1,2,4,5-Tetrazin-3-yl)benzyl)amino)-2-(4-((5-(2-((*tert*-butoxycarbonyl)amino)ethyl)-2-hydroxyphenyl)diazenyl)benzamido)-3-oxopropane-1-sulfonic acid **3b**

Crude **2b** (∼104 µmol, 1 eq) was dissolved in CH_2_Cl_2_ (2 mL) and DMF (0.5 mL), to which was added (4-(1,2,4,5-tetrazin-3-yl)phenyl)methanamine hydrochloride (25 mg, 114 µmol, 1.1 eq, Sigma), followed by EDCI (24 mg, 125 µmol, 1.2 eq) and DMAP (cat.). The reaction mixture was stirred at RT for 2 h and adjudged complete at this time by LC–MS analysis. The reaction mixture was acidified with 1 m HCl (10 mL) then extracted with 3:1 CHCl_3_/IPA (3 × 10 mL). The combined organic fractions were then washed with 1 m HCl (2 × 10 mL) and then brine (10 mL). The organics were dried (Na_2_SO_4_), filtered, and concentrated under vacuum. This gave the desired product **3b** that was used without further purification. The corresponding m/z as detected by LRMS via electrospray ionization is the following: *m*/*z* (ES−) 704 [M − H]^−^.

#### (*E*)-*N*-(2-((4-(1,2,4,5-Tetrazin-3-yl)benzyl)amino)-2-oxoethyl)-4-((5-(2-aminoethyl)-2-hydroxyphenyl)diazenyl)benzamide

Crude **3b** (∼104 µmol, 1 eq) was deprotected as for **3a**. The corresponding m/z as detected by LRMS via electrospray ionization is the following: *m*/*z* (ES+) 606 [M + H]^+^, *m*/*z* (ES−) 604 [M − H]^−^.

#### Tetrazine-diazobenzene-biotin TDB3

Crude amine (∼104 µmol, 1 eq) was dissolved in DMF (2 mL), to which was added NHS-PEG4-Biotin (73 mg, 125 µmol, 1.2 eq, Thermo Scientific EZ-Link NHS-PEG4-Biotin—21363) and Hünig's base (55 µL, 312 µmol, 3 eq). The reaction was monitored by LC–MS analysis and was adjudged complete after 2 h. The reaction mixture was directly purified by semi-preparative HPLC (10–90% MeCN in H_2_O over a 35-min gradient at 4 mL min^−1^ using Phenomenex Luna, 5µ, C18, 100 Å column). The product fractions were combined and freeze dried to give **TDB3** (10 mg, 10% yield over four steps) as a red/orange powder. The corresponding m/z as detected by LRMS via electrospray ionization is the following: *m*/*z* (ES+) 1,080 [M + H]^+^, *m*/*z* (ES−) 1,078 [M − H]^−^.

### Cell culture and lysate preparation

L cells were purchased from ATCC (CRL-2648), and the murine KPC mouse-derived PDAC cell line K8484 (27) was kindly provided by the laboratory of Duncan Jodrell. Both lines were cultured in DMEM/F-12, HEPES (Gibco 31330038), supplemented with 10% FBS (Gibco 10270106) at 37 °C and 5% CO_2_ atmosphere.

For SILAC, labeling cells were passaged for at least 10 days in DMEM/F12 for SILAC (Thermo Fisher Scientific 88370) supplemented with 10% dialysed FBS and the respective heavy- or light-labeled amino acids.

For lysate preparation, cells were washed with cold PBS, scraped off the plate in 0.5–1 mL PBS containing complete protease inhibitor cocktail (Roche) on ice, centrifuged at 500*g* for 3 min, and washed again with protease inhibitor containing PBS before storing cell pellets at −80 °C. Cell pellets were lysed in 25 mm HEPES pH 7.2, 8 m urea (denatured), or 25 mm HEPES pH 7.2, 150 mm NaCl (native), by sonication (Diagenode Bioruptor, high intensity, 10–20 cycles 30 s on, 30 s off at 4 °C). Lysates were cleared by centrifugation at 21,100*g* for 10 min at 4 °C and protein concentration determined using a BCA assay (PIERCE 23227) before storage at −80 °C.

### Generation of hCD81_Luc-expressing cell line

pLVX-Hygro-hCD81_Luc was transfected into HEK293T cells along with the third-generation packaging vectors (pMDL, pCMV-Rev, and pVSV-G) using Lipofectamine2000 (Thermo Fisher Scientific). Virus was collected, filtered (0.22 μm), and stored at −80 °C. A stable L cell line expressing hCD81_Luc was generated by infecting with lentiviral particles and selection with 500-µg/mL hygromycin B.

### Generation of SORT cell lines and proteomic incorporation of AlkK or CypK

For stable integration of the SORT, machinery cell lines were transfected in a 6-well plate with FuGENE HD (Promega) and 2 µg plasmid DNA, Super PiggyBac Transposase Plasmid (SBI), PiggyBac 4×U6-PylT(U25C)/EF1-*Mm*PylS-IRES-Puro, and -Blas in a ratio of 1:2:2. After 48 h, cells were split 1:6 into a 6-well plate each, and selection antibiotic was added. Optimal antibiotic concentrations (0.5–2 µg/mL puromycin, 1–5 µg/mL blasticidin) were experimentally determined. Cells were grown for at least 7 days under selection for polyclonal pools. Clonal cell lines were generated using serial dilution, and expression levels of *Mm*PylS were assessed by Western blotting normalizing to GAPDH.

For proteomic incorporation of the respective click amino acid, cells were grown in medium supplemented with either 1 mm AlkK (synthesized in house) or 0.25 mm CypK (Sirius fine chemicals SC-8017) for at least 72 h.

### Preparation of EVs from cell culture supernatant

For EV preparation from individual cell lines, cells were seeded in 150-mm dishes at 2–3E6 cells/mL and grown to 80–90% confluency in full growth medium. At this stage, the FBS-containing medium was aspirated and cells were washed with 3 × 10 mL PBS before the addition of 20 mL serum-free growth medium for 24 h (cancer cell lines) or 72 h (L cells). The conditioned cell culture supernatant was centrifuged at 300*g* for 5 min at RT, followed by 2,000*g* for 10 min and a final filtration step (0.22 µm) to remove debris and larger vesicles. Up to 50 mL or 200 mL of supernatant were concentrated 200× using MWCO 100-kDa filters Amicon Ultra-15 (Millipore UFC910024) or Centricon Plus-70 (Millipore UFC710008), respectively. For buffer exchange or removal of excess CypK or AlkK, samples were diluted with 10 volumes of 25 mm HEPES pH 7.2, 150 mm NaCl, and re-concentrated three times. EV samples were stored at 4 °C for up to 2 weeks or kept at −80 °C for long-term storage.

### Size exclusion chromatography

A total of 250–500 µL EV samples were fractionated using 10 mL Sepharose CL-2B (GE Healthcare 17-0140-01) gravity flow size exclusion columns (Bio-Rad Laboratories 732-1010) in 25 mm HEPES pH 7.2, 150 mm NaCl. A total of 0.5 mL fractions were collected, and EVs containing fractions F7–9 were used for downstream processing or analysis.

### EV affinity purification

EVs were affinity purified after ultrafiltration or size exclusion chromatography using antibodies targeting tetraspanin CD81 (anti-mouse CD81 Eat-2 BD Bioscience 559518, anti-human CD81 5A6 BioLegend 34514) or PS-binding protein Tim4 (produced in 293T as Fc fusion protein and in vivo biotinylated using AviTag system). Five-µg biotinylated antibody or Tim4 was immobilized on 20-µL streptavidin magnetic beads (PIERCE 88816). Preequilibrated beads were incubated with EV containing samples at 4 °C overnight in 25 mm HEPES pH 7.2, 150 mm NaCl, 0.05% Tween-20 supplemented with 2 mm CaCl_2_ in case of Tim4 capturing. Beads were washed 3× with 500 µL of buffer before further processing for downstream analysis.

### Chemoselective labeling of lysates and native EVs with tetrazine probes

CypK containing samples were reduced with 2.5 mm DTT for 1 h at RT, followed by alkylation with 15 mm iodoacetamide for 30 min at RT in the dark. Alkylated samples were then labeled with 20 µm of the respective tetrazine compound (6-methyl-tetrazine-sulfo-Cy5 Jena Bioscience CLK-1019-1, tetrazine-PEG4-biotin Jena Bioscience CLK-027-25, or tetrazine-diazobenzene-biotin derivatives synthesized in house). Urea lysates were labeled for 3 h at RT, while EV samples were labeled at 4° C overnight. Labeling reactions were quenched by the addition of 1 mm BCN-OH (Sigma-Aldrich 742678) for 10 min at RT before further processing or analysis.

### Chemoselective labeling of lysates and native EVs with azide probes

AlkK containing samples were reduced with 2.5 mm DTT for 1 h at RT, followed by alkylation with 15 mm IAA for 30 min at RT in the dark. Alkylated samples were then mixed with 100 µm of the respective azide compound (AF488/AF647-picolyl-azide Jena Bioscience CLK-1276-1/1300-1, biotin-PEG4-picolyl-azide Sigma 900912), supplemented with 1× copper-ligand mix (from 50× stock, final concentrations 500 µm THPTA, 250 µm CuSO4) before starting the reaction by the addition of 2.5 mm ascorbic acid. Urea lysates were labeled for 3 h at RT, while EV samples were labeled at 4 °C overnight. Labeling reactions were quenched by the addition of 1 mm AlkK for 10 min at RT before further processing or analysis.

### Luciferase-based extracellular vesicle recovery assay

Immobilization of EVs to white streptavidin-coated 96-well plates (PIERCE 15502) was performed in 100 µL of 25 mm HEPES pH 7.2, 150 mm NaCl, 0.05% Tween-20 either through SORT-enabled direct biotinylation of EVs or via biotinylated EV binding molecules (see EV affinity purification). After immobilization overnight at 4 °C, the plates were washed three times with 200 µL 25 mm HEPES pH 7.2, 150 mm NaCl, 0.05% Tween-20, and EV recovery was measured by NanoGlow luciferase assay (Promega N1120) and recovery normalized to the respective input signal.

For the analysis of luciferase recovery from prelysed EVs, concentrated EVs were incubated 25 mm HEPES pH 7.2, 150 mm NaCl, 0.5% NP-40 for 30 min at RT, before dilution to assay conditions.

Biotinylation of EVs for SIEVE was performed with concentrated EVs, and labeled EVs were diluted for binding to adjust for biotin binding capacity of the plates.

### SIEVE of cell culture-derived EVs

Biotinylation of SORT-labeled EVs derived from cell culture supernatant is performed in a total volume of 500 µL as described above. After quenching of the labeling reaction, the samples are subjected to size exclusion chromatography to remove excess free labels. EVs containing fractions F7–9 were supplemented with 0.05% Tween-20 and processed as described for EV affinity purification.

### MS sample preparation from bead-captured EVs

SIEVE-captured or affinity-purified EVs bound to magnetic beads were washed with 500 µL detergent-free buffer (25 mm HEPES pH 7.2, 150 mm NaCl, ±2 mm CaCl_2_), transferred to a fresh Protein LoBind tube in 100 µL detergent-free buffer, and lysed on beads with a mixture of 15 µL 0.2% ProteaseMAX (Promega V2017) and 20 µL 8 m urea in 50 mm ABC for 30 min at RT. The lysate was removed from the beads and volume adjusted to 100 µL with 50 mm ABC before reduction with 5 mm DTT for 30 min at RT followed by alkylation with 15 mm IAA for 20 min at RT in the dark. Before incubation with 0.25 µg trypsin (Promega V5113) at RT overnight, samples were supplemented with additional 5 mm DTT and 1 µL of 1% ProteaseMAX solution.

Trypsin digest was stopped by the addition of 0.5% TFA, and samples were further supplemented with 2% acetonitrile. Before desalting samples via C18 SPE stage tipping (33), ProteaseMAX degradation products were removed by centrifugation at 15,000*g* for 10 min at RT.

### MS sample preparation from EVs in solution

Ten-µg total protein for concentrated samples after ultrafiltration were supplemented with 1 µL of GlycoBlue in total volume of 50 µL in 2 mL tube Protein LoBind tube. The tube is filled with MS-grade ethanol at RT (>95% final EtOH concentration) and incubated at −80 °C overnight. The following day samples were spun at 18,000*g* for 1 h at 4 °C, the supernatant was aspirated, and pellets were dried at RT. Pellets were then resolubilized in 40-µL 100 mm ammonium bicarbonate (ABC), 8 m urea, and reduced with 5 mm DTT for 30 min at RT followed by alkylation with 15 mm IAA for 20 min at RT in the dark. Before incubation with 0.125 µg LysC for 4 h at RT (Wako Chemicals 125-05061), alkylation reaction was quenched by adding additional 5 mm DTT for 10 min at RT. After initial digest with LysC, samples were diluted to a final urea concentration of 2 m with 100 mm ABC and supplemented with 0.25 µg trypsin for overnight digest at RT.

### LC–MS

Peptide mixtures were separated by online reversed phase chromatography using the Dionex UltiMate 3,000 UHPLC system. Peptides were first loaded onto a C18 trap column (Acclaim PepMap 100: inner diameter, 100 μm; length, 2 cm; particle size, 5 μm; pore size, 100 Å) and then resolved on a 25-cm C18 analytical column (EASY-Spray: inner diameter, 75 μm; particle size, 2 μm; pore size, 100 Å) through a 120-min gradient of 1.6–32% acetonitrile plus 0.1% formic acid and 5% DMSO at a flow rate of 250 or 300 nL/min. Eluted peptides were sprayed directly into the Q Exactive HF quadrupole-Orbitrap mass spectrometer (Thermo Scientific).

Mass spectra were acquired in data-dependent mode using a top 10 sensitive method. Each duty cycle consisted of one MS full scan in the Orbitrap mass analyzer (resolution, 60,000 at *m*/*z* 200; scan range, 350 to 1,650 *m*/*z*; target value, 3 × 10^6^; maximum injection time, 60 ms) and subsequent MS/MS scans of 10 most intense precursor ions fragmented via higher energy collision dissociation (HCD; resolution, 60,000 at *m*/*z* 200; target value, 2 × 10^5^; maximum injection time, 120 ms; isolation window, 1.5 *m*/*z*; normalized collision energy, 27%). Precursor ions with unassigned, +1, +6, or higher charge state (+7 or higher for some samples) were not selected for fragmentation scans. Additionally, precursor ions already isolated for fragmentation were dynamically excluded for 30 s.

### MS data processing

MS raw data were processed using MaxQuant software (version 1.5.5.1) (34) and its Andromeda search engine (35). Mass spectra of fragment ions were searched against a concatenated target–decoy database containing the forward and reverse protein sequences of UniProt human (92,960 entries) and mouse (58,458 entries) proteomes release 2016_11 and a list of 245 common contaminants. Corresponding SILAC labels were selected for SILAC experiments. The proteome database was digested in silico based on LysC/P or trypsin/P specificity (cleave *C*-terminally to arginine or lysine residues even if followed by proline) and a maximum of two missed cleavages. Carbamidomethylation of cysteine was set as fixed modification. Both methionine oxidation and protein *N*-terminal acetylation were considered as variable modifications. Minimum peptide length was seven amino acids. The “Second peptides” option was also activated. For each protein group, at least one unique peptide was required. False discovery rate (FDR) was set to 1% at both peptide and protein levels. For protein quantification, both the unique and razor peptides were used. Minimum SILAC ratio count was set to one. The “re-quantify” function was switched off.

### SDS-PAGE and Western blotting of SORT-labeled lysates

Samples were supplemented with Laemmli sample buffer (Bio-Rad Laboratories) containing 50 mm DTT and incubated at 90 °C for 10 min. Reduction and heat denaturation were omitted for EV samples as well as urea-containing samples. Proteins were then resolved using 4–20% Mini-PROTEAN TGX gradient gels (Bio-Rad Laboratories) and transferred to a nitrocellulose membrane (Bio-Rad Laboratories). Blots were detected using IRDye 800CW conjugated streptavidin (Li-Cor 926-32230) according to the manufacturer's recommendations and detected using the Odyssey system. Staining for total protein load was performed with Ponceau-S.

### Statistics and data reporting

All statistical analysis were performed in the R (v4.0.0, https://www.r-project.org/) or Python (v3.7.6, http://www.python.org) software environments.

### Differential protein abundance analysis

Proteins with differences in abundance between conditions were identified using the “limma” R package (v3.44.1).

### GSEA

Preranked GSEA was used to identify proteins enriched on a pathway level. Proteins were preranked by log fold change derived from limma analysis. “fgsea” R package (v1.14.0) was used to identify enrichment of gene ontology cellular components: C5:CC gene collections from “msigdbr” R package (v7.1.1). Sets of proteins that were differentially enriched in the L cell and K8484 secretome were identified using limma. These proteins were then used with single sample GSEA (ssGSEA), using the “GSVA” R package (v1.36.1), to determine enrichment of K8484 and L cell gene sets in different cell culture conditions.

### Clustering

PCA was performed using base R statistics (v4.0.0, https://www.r-project.org/). Clustermaps were produced using seaborn (v0.10.0). Samples were clustered hierarchically by Euclidean distance.

## Supplementary Material

pgad107_Supplementary_Data

## Data Availability

All data collected as part of this study (MSMS raw files) will be submitted to the Proteomics Identifications database (PRIDE) https://www.ebi.ac.uk/pride/ at acceptance of manuscript, and a list of MSMS raw files used for each figure will be provided. There are no restrictions to the use of data and raw files from this study.

## Code availability

All code used in this work is available upon request.
