# Supplementary material for: Cell-of-origin–specific proteomics of extracellular vesicles
Source: PNAS Nexus. 2023 Apr 3;2(4):pgad107. doi: 10.1093/pnasnexus/pgad107 (PMC10119638; doi:10.1093/pnasnexus/pgad107)
Supplement: pgad107_Supplementary_Data [file pgad107_supplementary_data.zip › PNASNEXUS-PNASNEXUS-2022-00936-T-s05.docx]

# Supplementary Material

### Supplementary Figure S1


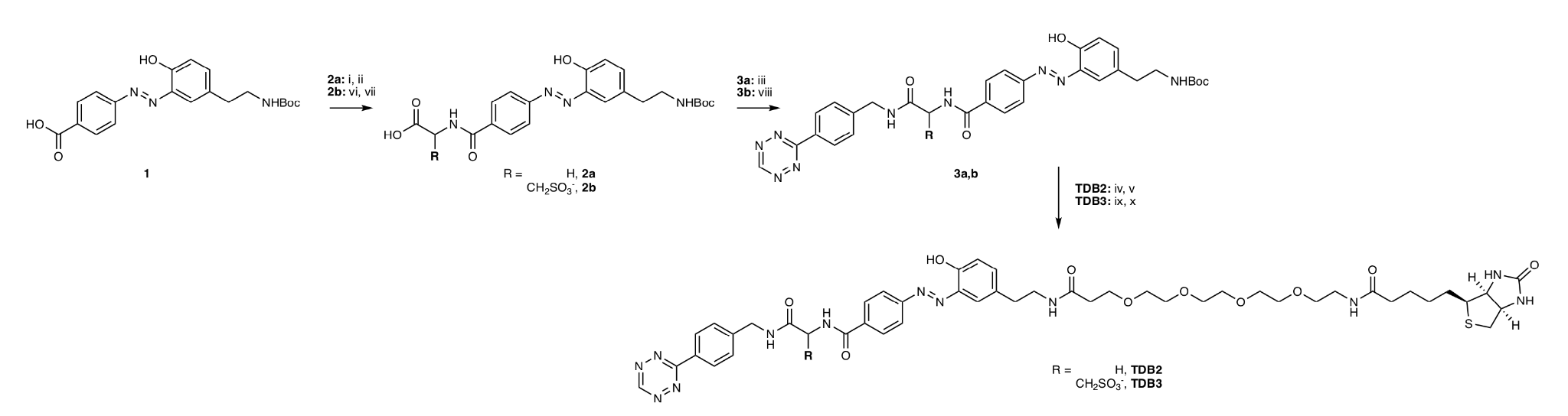


**Fig. S1.** Synthesis of TDB2 and TDB3. Reagents and conditions for **TDB2**: **i.** H-Gly-OMe, CH_2_Cl_2_, DMF, EDCI, HOBT, DMAP, RT, 20% yield; **ii.** LiOH, THF, H_2_O, RT, quantitative yield; **iii.** tetrazine-amine (Sigma), CH_2_Cl_2_, DMF, EDCI, DMAP, RT; **iv.** TFA, CH_2_Cl_2_; **v.** NHS-PEG4-Biotin (Thermo scientific), DMF, Hünig’s base, 15% yield over 3 steps. For **TDB3**: **vi.** *N*-hydroxysuccinimide, DMF, EDCI, DMAP, RT, 48% yield; **vii.** L-cysteic acid monohydrate, Hünig’s base, DMF, RT; **viii.** tetrazine-amine, CH_2_Cl_2_, DMF, EDCI, DMAP, RT; **ix.** TFA, CH_2_Cl_2_, RT; **x.** NHS-PEG4-Biotin (Thermo scientific), DMF, Hünig’s base, RT, 10% yield over 4 steps. For details on synthesis steps, see Methods.

**TDB2:**N-(2-((4-(1,2,4,5-tetrazin-3-yl)benzyl)amino)-2-oxoethyl)-4-((E)-(5-(4,20-dioxo-24-((3aS,4S,6aR)-2-oxohexahydro-1H-thieno[3,4-d]imidazol-4-yl)-7,10,13,16-tetraoxa-3,19-diazatetracosyl)-2-hydroxyphenyl)diazenyl)benzamide

**TDB3:**3-((4-(1,2,4,5-tetrazin-3-yl)benzyl)amino)-2-(4-((E)-(5-(4,20-dioxo-24-((3aS,4S,6aR)-2-oxohexahydro-1H-thieno[3,4-d]imidazol-4-yl)-7,10,13,16-tetraoxa-3,19-diazatetracosyl)-2-hydroxyphenyl)diazenyl)benzamido)-3-oxopropane-1-sulfonic acid

### Supplementary Figure S2

###
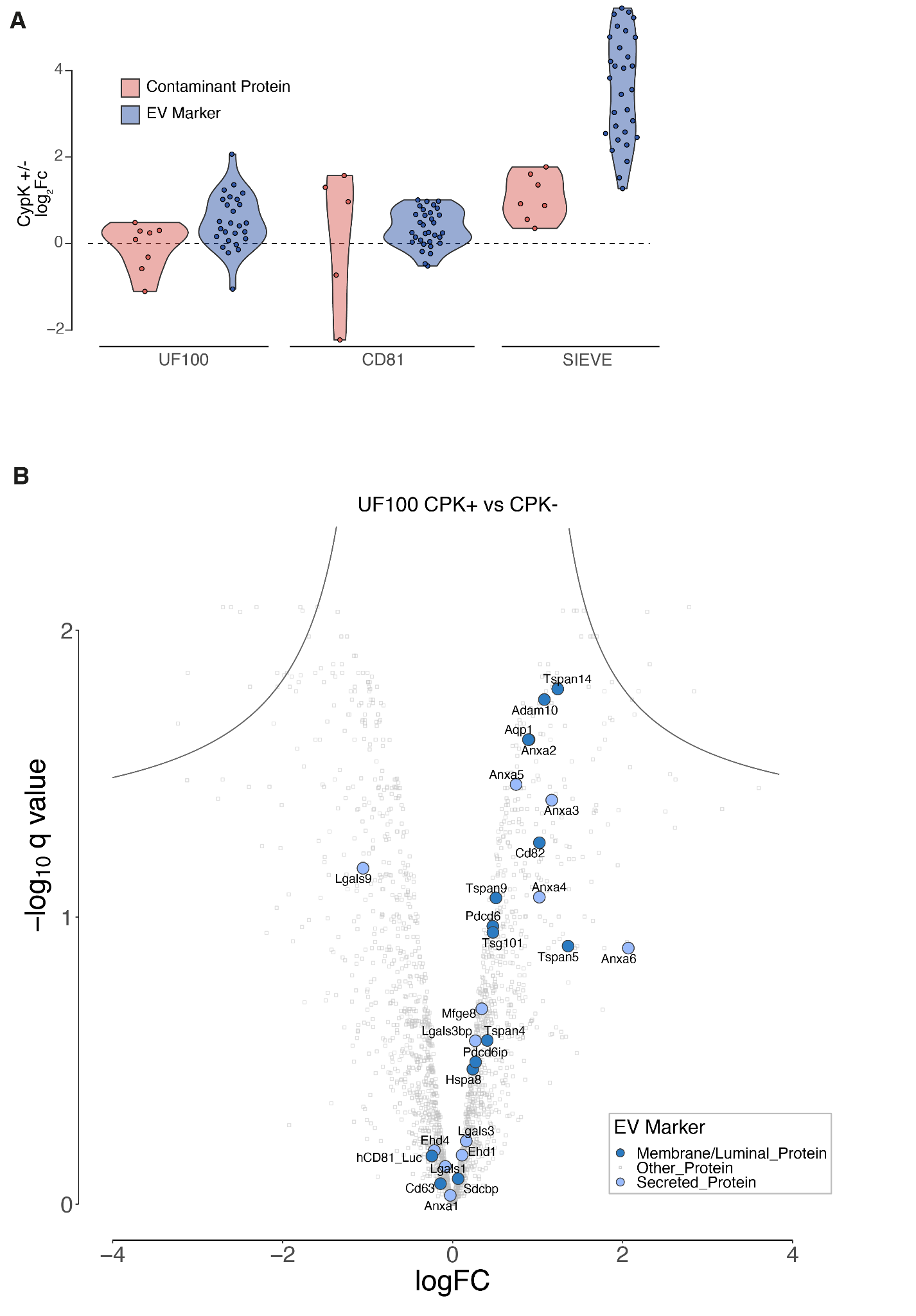


**Fig. S2.** Comparison of contaminant vs EV marker proteins between EV enrichment approaches. (**A**) Log2 fold change of EV marker and contaminant (see Table S1, Supplementary Material) protein abundances in CypK^+^ and CypK^-^ conditions from L cell_SORT_GCU_-hCD81_NLuc_ cells enriched for EVs with three approaches as indicated (data relating to Figure 3). Note that SIEVE selectively enriches for EV marker proteins but not contaminant proteins providing a means to distinguish EV proteins (beyond canonical marker proteins) from background proteins (beyond contaminants) based on the CypK^+^/CypK^-^ ratio, which is not directly possible with the other methods. (**B**) Differential abundance analysis of UF100 enrichment of CypK^+^ EV proteins over CypK^-^ control expressed as log_2_ fold change (log_2_FC) (data from Figure 3) and significance of change using multiple hypothesis corrected p value from two sided t tests (q value). Note that no canonical EV marker proteins (and a limited number of proteins in general) change significantly between conditions if analyzed by a non-SORT method (here UF100). Thus, even if cells incorporate ncAAs in their proteome (CypK^+^), canonical EV marker protein abundance does not differ significantly to cells that have not incorporated ncAAs (CypK^-^), indicating limited perturbation effects of the SORT system to EV marker proteins.

### Supplementary Figure S3

**
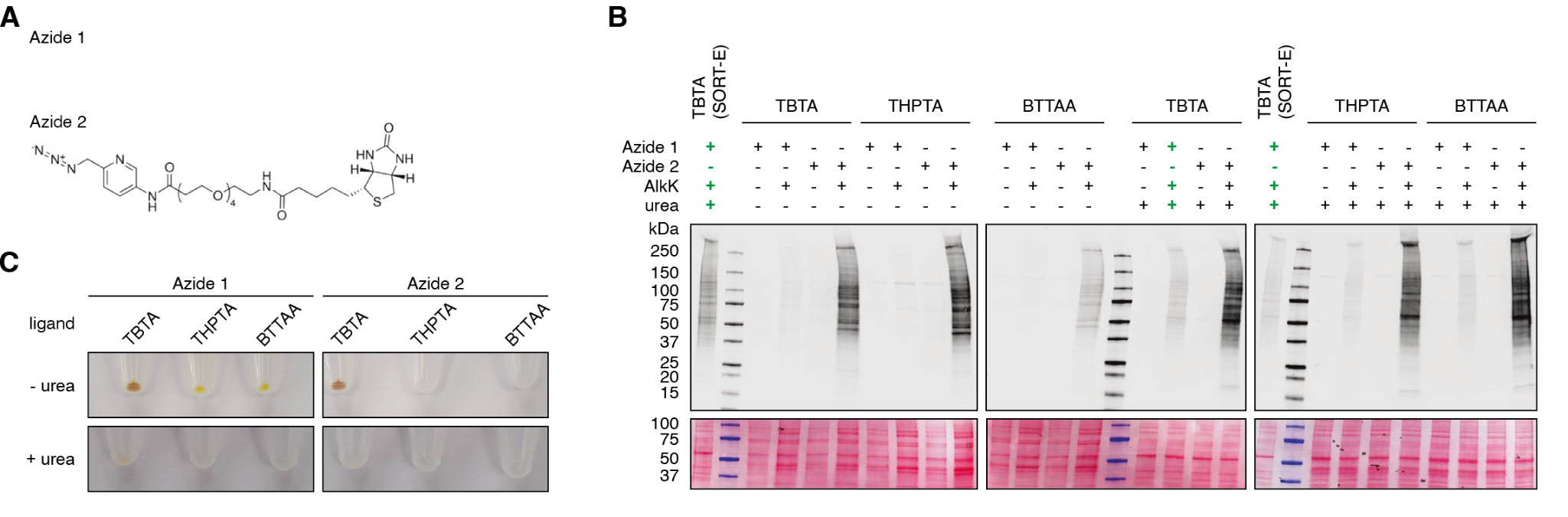
**

**Fig. S3.** Optimization of CuAAC labeling conditions for improved labeling efficiency under non-denaturing conditions. (**A**) Chemical structures of the cleavable azide-diazobenzene-biotin (azide 1) previously used for SORT-E and the non-cleavable picolylazide-PEG_4_-biotin. (**B**) L cell_SORT_GCU_-hCD81_NLuc_ lysate labeling comparing the two different azides and three different copper ligands (TBTA - Tris[(1-benzyl-1H-1,2,3-triazol-4-yl)methyl]amine, THPTA - Tris-(3-hydroxypropyltriazolylmethyl)-amine, and BTTAA - 2-(4-((Bis((1-(tert-butyl)-1H-1,2,3-triazol-4-yl)methyl)amino)methyl)-1H-1,2,3-triazol-1-yl)acetic acid) under native and urea conditions. Biotinylated proteins were separated by SDS-PAGE and detected by Western blotting using IR-dye labeled streptavidin. Ponceau-S stained membranes shown for loading control. Previously used SORT-E conditions are highlighted and indicated by green symbols. (**C**) Photograph of precipitates after the labeling reaction.

###

### Supplementary Figure S4

**
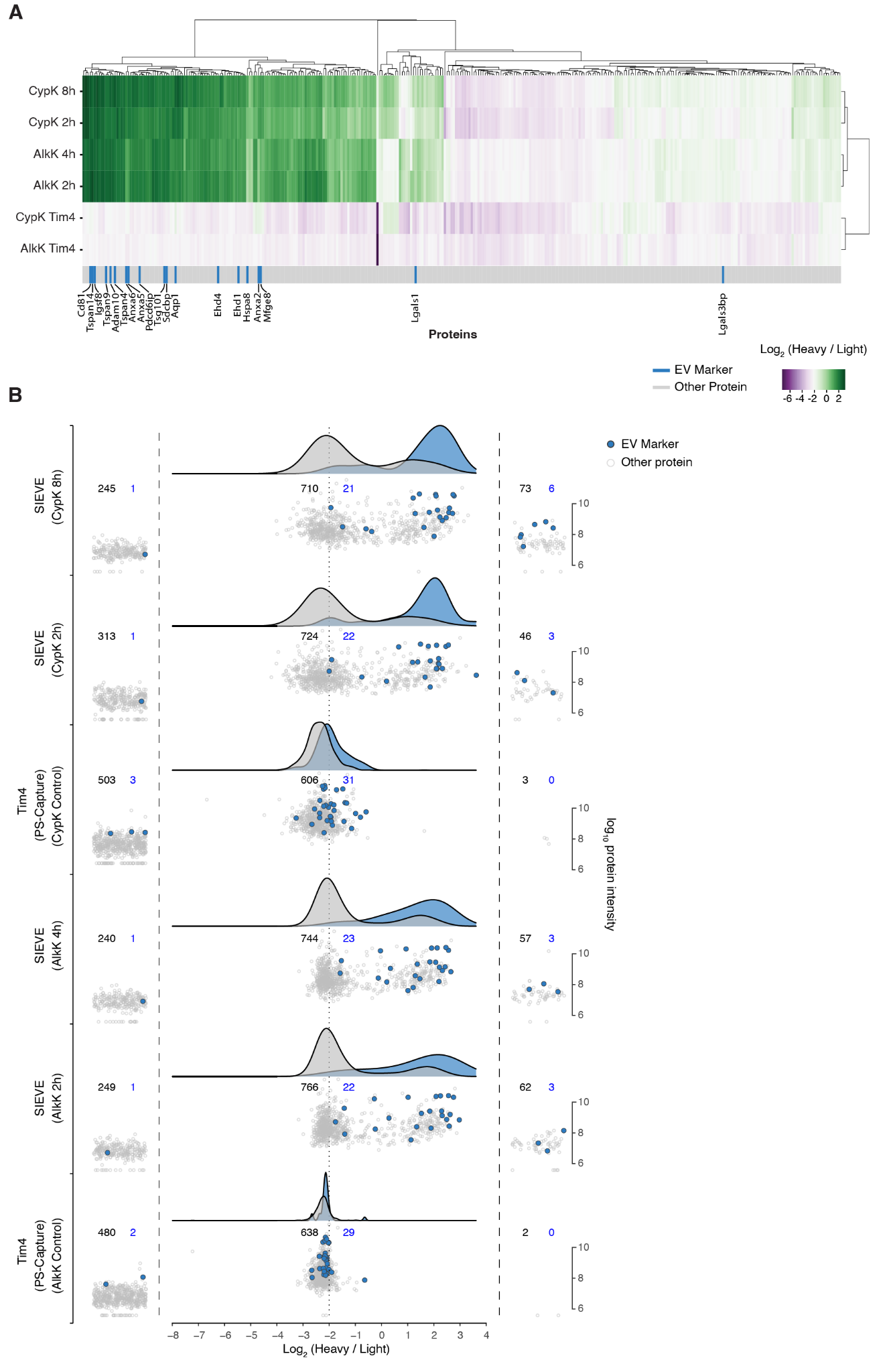
**

**Fig. S4.** Low-abundant EV subpopulations are selectively enriched by SIEVE irrespective of the labeling chemistry. (**A**) Hierarchical clustering using Euclidean distance of log_2_(H/L ratios) obtained for EV isolation by SIEVE using SPIEDAC (CypK) and CuAAC (AlkK) at two different labeling durations each (CypK - 2h and 8h, AlkK - 2h and 4h) and their respective Tim4 control (PS-capture). Position of EV marker proteins are indicated by blue bars (n = 338 proteins quantified across all six conditions). (**B**) Distribution of the H/L ratio for all quantified proteins comparing isolation by SIEVE using either CypK incorporation followed by SPIEDAC labeling with tetrazine-biotin or AlkK incorporation and CuAAC labeling with azide-biotin, and generic isolation by Tim4 (PS-capture). PS-capture control has been performed with both inputs and SIEVE labeling has been performed with two different labeling durations each. EV marker proteins are highlighted in blue and density plots of H/L ratio distributions of marker and non-marker proteins are shown on top using the same color scheme. Number of quantified proteins are given for shared, light only and heavy only populations.

### Supplementary Figure S5

**
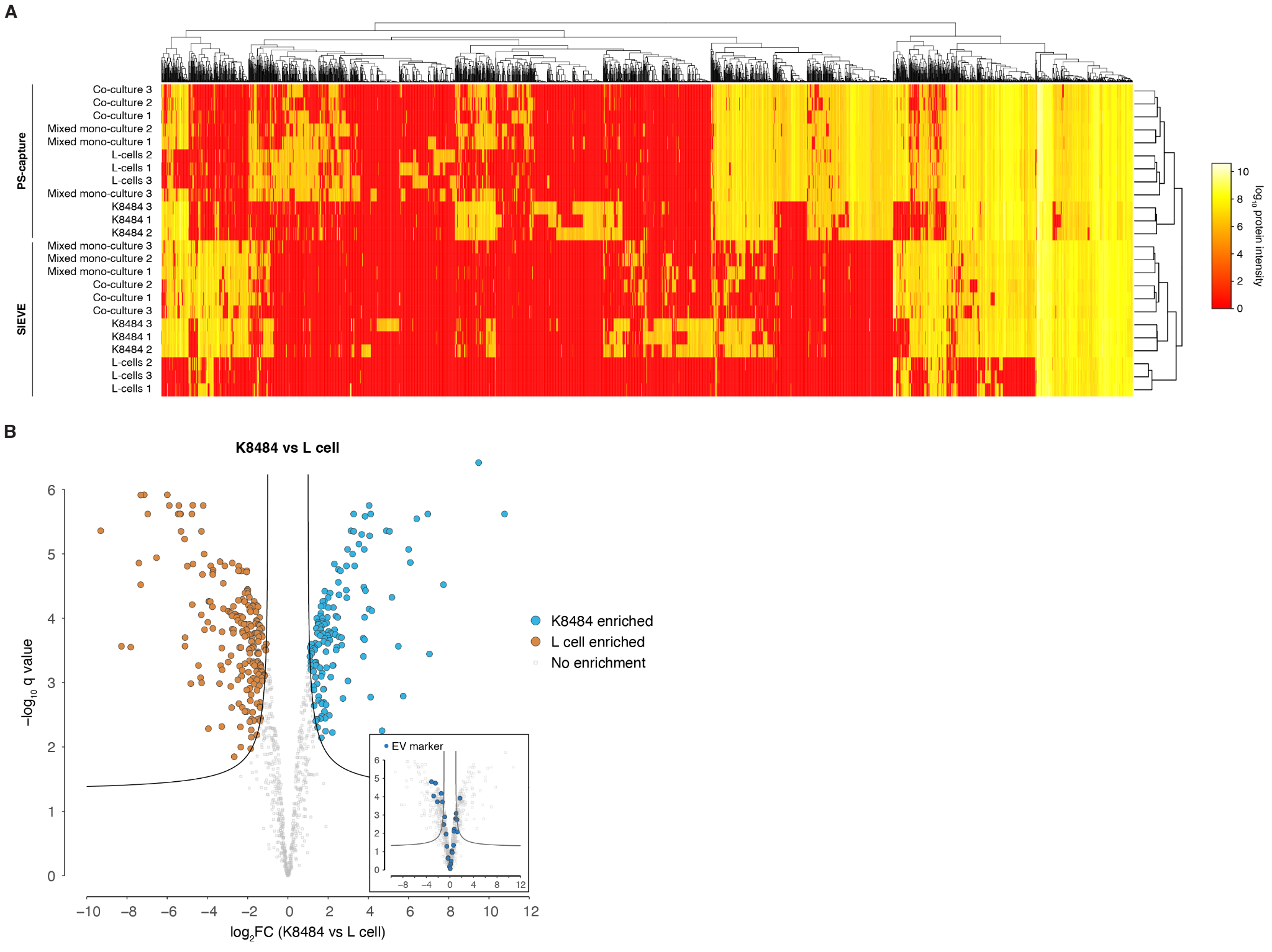
**

**Fig. S5.** Target cell derived EVs are selectively enriched from co-culture allowing cell type selective EV proteome analysis. (**A**) Proteome clustermap of log_10_ intensities. Conditions and proteins clustered hierarchically based on euclidean distance. Only proteins detected in at least one condition included (n = 3167). Red color indicated not detected or quantified. Note that in the L cell monocultures analyzed by SIEVE (bottom three rows) comparatively few proteins are identified as expected. (**B**) Differential expression analysis of PS-captured EV samples by Tim4 of K8484_SORT_GCU_ cells against L cells expressed as log_2_ fold change (log_2_FC) and significance of change using multiple hypothesis corrected p value from two sided t tests (q value). Inset shows differential expression of shared EV marker proteins highlighted as blue circles. Lines represent the cut-off curve for significance (S0 = 1, q < 0.05).

### Supplementary Table S1

**List of EV marker and general EV-related contaminant proteins.** EV maker proteins are derived from literature [(1, 3, 25)](https://paperpile.com/c/pMOhRO/wI6K7+LXFCs+wzRbh) and MS analysis of EV proteomes of multiple cell lines (unpublished data). List of general contaminant proteins commonly identified in plasma EV pulldown experiments.

### Supplementary Table S2

**List of L cell proteins identified and differential abundance analysis in CypK+ and CypK- conditions across three EV analysis methods (relating to Figure 3c).** Columns are: a) protein/gene name, b) log_2_ fold changes between CypK+ and CypK- condition, c) mean log2 expression across conditions, d) moderated t-statistic, e) p-value, f) FDR adjusted p-value, g) B-statistic

### Supplementary Table S3

**Protein abundance across culture conditions and replicates (relating to Figure S5a).** Log_10_ intensities for proteins (gene name) across replicated (“_1”, “_2”, “_3”) in co-cultures (“Co”), mono cultures (“Lcell” and “K8484”) and mixed monocultures (“MM”) for the two enrichment experiments (SIEVE and PS-capture). Boolean annotations of whether each protein showed enrichment in each of the cell line has been included.

**Supplementary Table S4**

**List of protein differential abundance in K8484 and L-cells following CD81 capture (relating to Figure S5b).** Columns are: a) index b) log fold change c) mean log2 expression across conditions, d) moderated t-statistic, e) p-value, f) FDR adjusted p-value, g) B-statistic, h) protein/gene name, i) Ev marker information, j) annotation of enrichment in each cell line.
